# Long-term Surgical Outcomes of Epiretinal Membrane in Patients with Retinitis Pigmentosa

**DOI:** 10.1038/srep13078

**Published:** 2015-08-13

**Authors:** Yasuhiro Ikeda, Noriko Yoshida, Yusuke Murakami, Shunji Nakatake, Shoji Notomi, Toshio Hisatomi, Hiroshi Enaida, Tatsuro Ishibashi

**Affiliations:** 1 Department of Ophthalmology, Graduate School of Medical Sciences, Kyushu University, Fukuoka 812-8582, JAPAN; 2 Department of Ophthalmology, Saga University Faculty of Medicine, Saga, JAPAN

## Abstract

Macular complications such as an epiretinal membrane (ERM), a cystoid macular edema and a macular hole lead to unexpected central vision impairment especially for patients with retinitis pigmentosa (RP). To evaluate the long-term surgical outcomes of pars plana vitrectomy (PPV) for ERM in patients with RP, we retrospectively reviewed the charts of a consecutive series of 10 RP patients who underwent PPV for ERM at Kyushu University Hospital. Visual acuity (VA) testing, a fundus examination, and an optical coherence tomography (OCT) analysis were conducted. The standard PPV using three sclerotomies was performed for ERM. PPV was performed in 12 eyes of 10 patients. One eye was excluded from the outcome assessment due to short period observation (18 months). There was no significantly deleterious change from the baseline to final VA between the operation eyes and the fellow eyes (P = 0.19). Moreover, morphological improvement was obtained in 9 of 11 eyes based on OCT. Our present data suggest that PPV may be tolerable in the management for ERM in RP patients over the long-term. Furthermore, the appearance of the ellipsoid zone was an important factor in the prediction of visual outcome and determination of surgical indication.

Retinitis pigmentosa (RP), a genetically heterogeneous group of retinal degenerative diseases that affect photoreceptor and retinal pigment epithelial function, is a major cause of blindness in adults^1^,^2^. The prevalence of this disease has been estimated as approximately 1 in 4,000 people pan-ethnically^3^,^4^. Although most cases of typical RP are described as having a similar phenotype of equatorial bone-spicule pigment figures and attenuated retinal vessels along with rod-cone degeneration, the clinical features of this disease vary markedly among individuals. Macular complications such as the development of an epiretinal membrane (ERM), cystoid macular edema (CME) and a macular hole (MH) lead to unexpected central vision impairment^5^,^6^. The reported prevalence in RP cases of ERM including vitreomacular traction syndrome is 1.4%–20.3%^6^,^7^,^8^, that of CME is 10%–40%^5^,^7^,^9^, and that of MHs is 0.5%–10%^8^,^10^.

There have been a few reports of vitrectomy for these macular complications in patients with RP and their surgical outcomes have not all been favorable^8^,^11^. It has been reported that intraocular direct light and mechanical direct effect on the retina might further damage the inner and outer retina layers and a worsening of visual acuity might be associated with an increase of retinal thickness^12^. However, there have been few reports of long-term surgical outcomes; thus, the clinical effect of surgical treatment, including the effect on the retinal degeneration process, is still unclear.

We performed a retrospective observational clinical study with RP patients and assessed the functional and morphological long-term outcomes of pars plana vitrectomy (PPV) for ERM.

## Results

### Visual acuity

PPV was performed in 12 eyes of 10 patients (6 males and 4 females) at Kyushu University Hospital. One eye, that is the left eye of patient number (Pt no.) 2, was excluded from the outcome assessment due to short period observation (18 months). The clinical data are summarized in Tables 1 and 2. The mean age at which the PPV was performed was 50.6 ± 16.5 years (yrs) (range 24–73 yrs) and the mean follow-up time was 68.2 ± 12.3 months (mos) (range 51–86 mos). Cataract surgery was performed simultaneously in 5 of the 11 eyes that underwent PPV (45.5%). We detected MH post-PPV only in one eye (Pt no. 4). We also compared the surgical outcomes between the operation eyes and fellow eyes (Table 3).

The mean baseline best-corrected visual acuity (BCVA) score was 0.43 ± 0.27 (range 0.10–1.05) (Table 1). The mean postoperative (6 mos after) BCVA score was 0.36 ± 0.28 (range –0.08–0.70), and the mean final BCVA was 0.52 ± 0.83 (range –0.08–2.90). The final BCVA score was improved (0.3 logMAR or more) in 3 eyes (27.3%; the right eye of Pt no. 2, Pt no. 5 and the left eye of Pt no. 10), was unchanged in 6 eyes (54.5%), and worsened (0.3 logMAR or more) in 2 eyes (18.2%; Pt no. 3 and 6) compared with the baseline BCVA score. In one eye of the 2 worsened eyes, posterior capsule opacification occurred and a YAG laser capsulotomy will be needed (Pt no. 6). Moreover, both eyes of Pt no. 3 had no perception of light at all; thus, the final BCVA of the fellow eye also worsened.

In the comparison of the BCVA between the operation eyes and fellow eyes (Table 3), we excluded the eyes that underwent PPV to both eyes (Pt no. 10), and in which the final lens condition (phakic or pseudophakic eye) was different (Pts no. 2 and 6). In the statistical analysis of the final BCVA, there was no significant difference between the operation and fellow eyes (P = 0.795), though there was significant difference in that of baseline (P < 0.05). In addition, there was no significant difference in changes from the baseline to final BCVA between the operation eyes and the fellow eyes (P = 0.19). Furthermore, there was no significant correlation between the final BCVA and the final central subfield thickness (CST) in the operation eyes (r = −0.36, P = 0.31), excluding Pt no. 4 (the post-PPV MH formation eye).

### Central subfield thickness

The data of CST and the grading for appearance of the ellipsoid zone (EZ; also called the inner/outer segment [IS/OS] junction) at the fovea from optical coherence tomography (OCT) images are shown in Table 2^13^,^14^. Baseline data was not obtained for all eyes due to unavailability for measurement. Posterior vitreous detachment was detected only in one eye at the baseline examination (Pt no. 1).

In the comparison of the CST between the operation eyes and fellow eyes (Table 3), we excluded the eyes that underwent PPV to both eyes (Pt no. 10), and which detected MH post-PPV (Pt no. 4). The mean of the final CST of post-PPV eyes was 228.1 ± 53.0 μm. The final CST in the operation eyes was no significantly difference compared with the fellow eyes (P = 0.066).

### Ellipsoid zone

**Figure 1:**
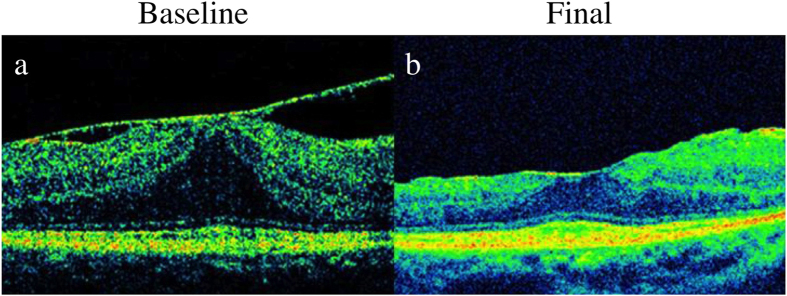
Images of horizontal optical coherence tomography macular scan from the typical cases. The baseline (**a**) and final (**b**) image post-pars plana vitrectomy of a 57-year-old Japanese woman (patient number 5) with epiretinal membrane.

We detected the EZ was not visible post-PPV only in one eye (the right eye of Pt no. 10), in addition to the post-PPV MH formation eye (Pt no. 4). Therefore, in nine of 11 eyes (81.8%), morphological improvement was observed post-PPV via OCT examination (Fig. 1a,b).

**Figure 2:**
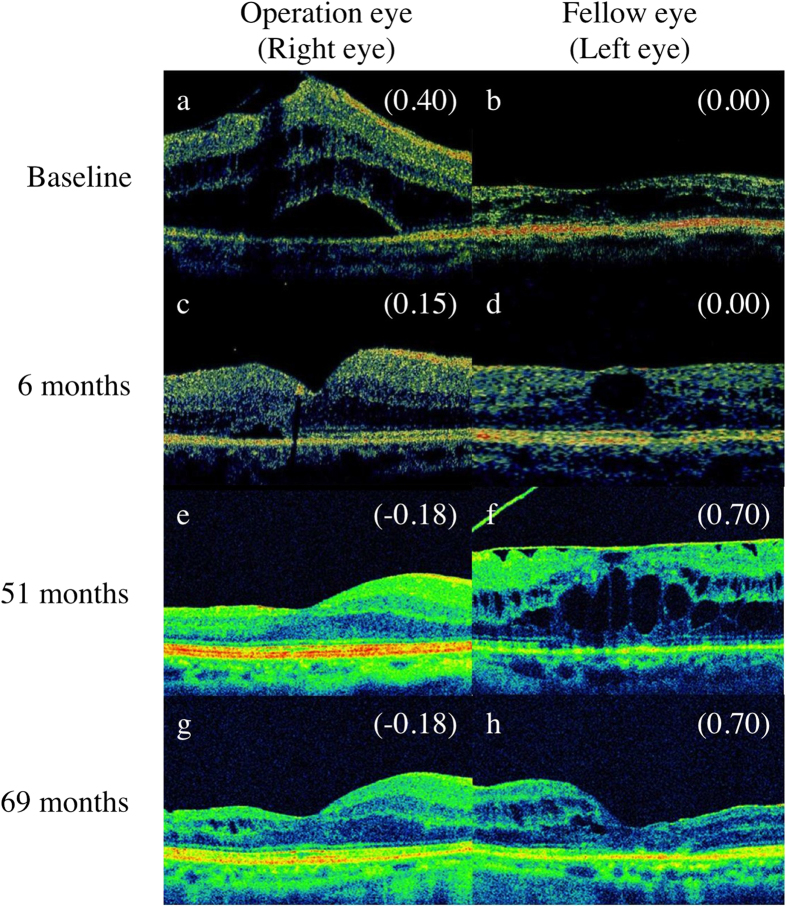
Images of horizontal optical coherence tomography macular scan from patient number 2. The images mainly show the ellipsoid zone status in both eyes. (**a**,**b**) The baseline scan. (**c**,**d**) At 6 months post-pars plana vitrectomy (PPV). (**e**,**f**) At approximately 4 years post-PPV (51 months). (**g**,**h**) At 18 months post-PPV for the left eye (69 months).

In case of Pt no. 2, the ERM was also observed in both eyes at the baseline visit (Fig. 2a,b). We performed PPV with cataract surgery only for right eye (the operation eye) because of decreased vision and the visual acuity was improved (Fig. 2c,e,g). On the other hand, we did not perform PPV for the left eye (the fellow eye) at that time because of good vision. At 51 mos post-PPV, we detected the progressive ERM and decreased vision in the fellow eye (Fig. 2d,f). We performed PPV with cataract surgery for the fellow eye, however, the visual acuity was unchanged at 18 mos post-PPV (Fig. 2h).

## Discussion

Macular complications such as ERM, CME and MH are particularly important because of their effect on central visual function. The first-line therapeutic intervention for ERM has been PPV with internal limiting membrane (ILM) peeling. However, surgical adverse effects of retinal damage including photo-toxicity during PPV should be considered in patients with RP. In the present retrospective study, we investigated the functional and morphological long-term outcomes of PPV for ERM with patients with RP. We demonstrated the following: (1) there was no significant difference in changes from the baseline to final BCVA between the operation eyes and the fellow eyes; (2) in nine of 11 eyes (81.8%), morphological improvement was observed post-PPV via OCT examination; (3) the appearance of the EZ was an important factor in the prediction of visual outcome and determination of surgical indication.

As shown in Tables 1 and 2, the surgical outcomes varied among the patients. The final BCVA score was improved (0.3 logMAR or more) in 3 eyes, and was unchanged in 6 eyes, compared with the baseline BCVA score. Though the final BCVA score was worsened (0.3 logMAR or more) in 2 eyes, the operation-related decreased vision was not observed. Moreover, there was no significant difference in changes from the baseline to final BCVA between the operation eyes and the fellow eyes. Cataract surgery was performed simultaneously in 5 eyes that underwent PPV. In the three cases with improved final BCVA, Pt no. 2 and 5 underwent simultaneous cataract surgery with PPV. In both cases, even though mild lens opacity was observed in the both treated and fellow eyes at the baseline, the lens opacity of the treated eyes was almost the same as that of the fellow eyes without decreased BCVA (the left eye of Pt no. 2: 0.00, the left eye of Pt no. 5: −0.18, respectively). It is undeniable that combined cataract surgery influence for the visual outcome of PPV, however, we considered that there was no apparent influence for the visual outcome in the present study. Further study is needed to clarify the influence of combined cataract surgery with PPV for ERM.

In almost all cases, morphological improvement was obtained by PPV with ILM peeling (81.8%) based on OCT. There was only eye (the right eye of Pt no. 10) in our series suspected of possible operation-related effects (disruption of the EZ). There was significant difference between the baseline and the final CST in the statistical analysis of the operation eyes as shown in Table 3 (P < 0.05). In the general population, there have been various reports about the influence of macular thickness on visual outcome after vitrectomy for ERM^15^,^16^. It has been also reported that the retinal thickness decreases rapidly after ERM surgery and the final thickness is expected to be greater than in normal eyes^17^. In RP patients, it has been reported that the foveal thinning appeared post-PPV^8^. In the present study, the final CST in the operation eyes became thinner. However, there was no significant difference between the operation eyes and the fellow eyes in the analysis of final CST post-PPV. In addition, there was no significant difference between the operation eyes and the fellow eyes in the analysis of final BCVA and no correlation between the final CST and the final BCVA in the operation eyes. Although the retinal thickness decreased after ERM surgery, it was still unclear the influence on visual outcome in RP patients.

MH formation was detected in one case with ERM post-PPV (Pt no. 4). The development of secondary MH after vitrectomy is rare, and to the best our knowledge, it has not been reported previously among RP patients^18^. In the general population, foveoschisis and the EZ defect have been reported as risk factors for the development of MH post-PPV^19^. In this case, foveoschisis and the EZ defect were detected at the baseline OCT examination. The indications of vitrectomy for RP patients need to be considered carefully. The presence of preoperative normal EZ may be an important factor to assure good surgical outcomes in RP patients.

Regarding the preoperative EZ status, we should concentrate the intriguing results in case of Pt no. 2. As shown in Fig. 2 and Tables 1 and 2, his baseline EZ appearance was normal in both the operation and fellow eyes and the ERM was also observed in the fellow eyes. After approximately 4 years, the EZ status was maintained in the operation eye (Fig. 2e). On the other hand, we detected the progressive ERM with abnormal EZ appearance and decreased vision in the fellow eye (Fig. 2f). We performed PPV for the fellow eye, however, the EZ appearance was not improved and the visual acuity was unchanged at 18 months post-PPV (Fig. 2h). There were many reports about the correlation of preoperative outer retinal microstructure and visual acuity post-PPV^20^,^21^,^22^,^23^. Functional damage of the photoreceptor due to long-standing inward traction led to the visual acuity reduction in patients with ERM. There is possibility that we missed the opportunity of proper treatment for the fellow eye in case of Pt no. 2.

In conclusion, morphological improvement was obtained by PPV with ILM peeling in almost all cases of ERM. The appearance of the EZ was an important factor in the prediction of visual outcome and determination of surgical indication. Our present data suggest that PPV may be feasible and tolerable in the management for ERM in patients with RP over the long term.

## Methods

### Patients

This retrospective study included a consecutive series of 10 RP patients who underwent PPV for ERM at Kyushu University Hospital (Fukuoka, Japan). The diagnosis of RP was based on the patient’s history of night blindness, side vision restriction, and marked reduced or non-recordable a- and b-wave amplitudes on electroretinogram testing, in addition to ophthalmoscopic findings (i.e., characteristic fundus changes in the attenuated retinal vessels and bone-spicule-like pigment clumping). We excluded any patient with uveitis or any disease that could cause RP-like fundus changes.

The investigation was carried out under approval from the Institutional Review Board of the Kyushu University Hospital, and was conducted in accordance with the tenets of the Declaration of Helsinki on biomedical research involving human subjects. The review board waived the need for written informed consent, because the study design comprised a retrospective chart review.

### Ophthalmic Data Collection

BCVA was measured for all patients with full subjective refraction using a Landolt ring chart at 5 m in decimal units. The decimal acuities were converted into a logarithm of the minimum angle of resolution (logMAR) for the statistical evaluation.

Slit-lamp biomicroscopy of the anterior segment and fundoscopic examination by both direct and indirect ophthalmoscopy are carried out routinely on all patients. We observed the details of lens, vitreous, and fundus findings. OCT is a well-recognized method of analyzing the retinal architecture, and it has been used for the diagnosis and monitoring of RP^24^,^25^,^26^. OCT (Cirrus HD-OCT model 4000, Carl Zeiss, Dublin, CA, USA: macular cube mode, sliced 512 × 128 cuts) demonstrated ERM. The macular information was not obtained for all eyes, due to cataract formation or patient unavailability for measurement.

Research software version 3.0 (Carl Zeiss Meditec, Inc.) was used to measure CST. All OCT images were also used for the identification and measurement of the EZ, with a distinct and continuous line indicating normal alignment of the membranous discs in the photoreceptor outer segments^13^,^14^. We graded the appearance of the EZ at the fovea from 1 to 3^26^: Grade 1, EZ was not visible; Grade 2, abnormal EZ; and Grade 3, normal EZ.

### Surgical procedure of pars plana vitrectomy

PPV was performed in 12 eyes of 10 patients (6 males and 4 females) at Kyushu University Hospital. First, the standard phacoemulsification was performed for phakic eyes with cataract. The surgical treatment was mainly performed by a standardized pars plana vitrectomy with triamcinolone acetonide (Kenacort-A; Bristol Pharmaceuticals KK, Tokyo, Japan) solution. Thereafter, brilliant blue G dye solution (0.25 mg/ml) prepared by dissolving brilliant blue G 250 (Sigma-Aldrich, St. Louis, Mo., USA) was used for ILM peeling^27^,^28^. Finally, an intraocular lens was inserted. After the conjunctiva was closed, gentamicin was injected subconjunctivally. The surgical outcome evaluation included the eyes underwent PPV and observed for 3 years or longer.

### Statistical Analysis

The data are presented as the arithmetic mean values ± standard deviation. All statistical analyses were performed using Wilcoxon signed rank test to compare the baseline and final examination, and Mann Whitney U test to compare the operation and fellow eye. P-values less than 0.05 were considered significant.

## Additional Information

**How to cite this article**: Ikeda, Y. *et al.* Long-term Surgical Outcomes of Epiretinal Membrane in Patients with Retinitis Pigmentosa. *Sci. Rep.* **5**, 13078; doi: 10.1038/srep13078 (2015).

## Figures and Tables

**Table 1:** The surgical outcomes of pars plana vitrectomy (BCVA).

Pt no.	Eye	Gender	Age at Surgery (yrs)	Follow-up Time (mos)	BCVA (logMAR)						Cataract Surgery
					Baseline		6 mos after		Final		
					R	L	R	L	R	L	
1	R	F	57	51	**0.10**	0.00	−**0.08**	0.00	−**0.08**	0.00	+
2	R	M	53	51	**0.40**	0.00	**0.15**	0.00	−**0.18**	0.70	+
3	L	M	73	54	0.52	**0.52**	0.40	**0.70**	2.90	**2.90**	−
4	L	M	56	56	0.15	**0.40**	0.22	**0.40**	0.40	**0.52**	−
5	R	F	57	70	**0.70**	−0.18	**0.30**	−0.08	−**0.08**	−0.08	+
6	L	M	73	71	0.15	**0.22**	0.10	**0.52**	0.22	**0.52**	+
7	L	M	38	74	0.05	**0.15**	0.00	**0.15**	0.00	**0.15**	−
8	L	M	65	79	−0.08	**0.22**	0.00	−**0.08**	0.00	**0.00**	+
9	R	F	24	80	**1.05**	0.15	**0.52**	0.10	**1.05**	0.05	−
10	L	F	30	86	0.40	**0.70**	0.40	**0.70**	0.52	**0.40**	−
	R	F	31	78	**0.30**	0.70	**0.70**	0.70	**0.52**	0.40	−

Pt no. = Patient number; yrs = years; mos = months; BCVA = best-corrected visual acuity; logMAR = logarithm of the minimum angle of resolution; R = right; L = left; F = female; M = male.

The bold font indicates the operation eyes.

**Table 2:** The surgical outcomes of pars plana vitrectomy (OCT).

Pt no.	Eye	Gender	Age at Surgery (yrs)	Follow-up Time (mos)	PVD	Central Subfield Thickness (μm)				Grading for the EZ appearance			
						Baseline		Final		Baseline		Final	
						R	L	R	L	R	L	R	L
1	R	F	57	51	+	**552**	360	**206**	365	**3**	3	**3**	3
2	R	M	53	51	−	**/**	**284**	561	**3**	3	**3**	2	
3	L	M	73	54	−	213	**336**	244	**207**	1	**1**	1	**1**
4	L	M	56	56	−	548	**563**	402	**86**	2	**1**	2	**MH**
5	R	F	57	70	−	**370**	279	**257**	255	**3**	3	**3**	3
6	L	M	73	71	−	277	**240**	3	**3**	3	**3**		
7	L	M	38	74	−	267	**376**	220	**197**	3	**3**	3	**3**
8	L	M	65	79	−	307	**299**	3	**3**	3	**3**		
9	R	F	24	80	−	**418**	244	**135**	261	**1**	3	**1**	3
10	L	F	30	86	−	454	**473**	166	**164**	2	**1**	1	**1**
	R	F	31	78	−	**525**	209	**166**	164	**2**	1	**1**	1

OCT = optical coherence tomography; Pt no. = Patient number; yrs = years; mos = months; PVD = posterior vitreous detachment; EZ = ellipsoid zone; R = right; L = left; F = female; M = male; MH = macular hole.

“/” = unknown data.

Grading for the EZ appearance = Grade 1, EZ was not visible; Grade 2, abnormal EZ; and Grade 3, normal EZ.

The bold font indicates the operation eyes.

**Table 3:** Comparisons of the retinal central subfield thickness and the BCVA between the operation eyes with ERM and the fellow eyes.

	Operation eyes (mean ± SD)	Fellow eyes (mean ± SD)	*P* values
BCVA (n=7)			
Baseline	0.45 ± 0.34	0.09 ± 0.23	0.034
Final	0.64 ± 1.08	0.47 ± 1.08	0.795
*P* values	0.813	0.250	
Central subfield thickness (n=8)			
Baseline	410.4 ± 84.3	272.6 ± 55.0	0.021
Final	228.1 ± 53.0	311.3 ± 110.2	0.066
*P* values	0.010	0.812	

BCVA = best-corrected visual acuity; SD = standard deviation.
